# The Impact of Smoking on Psoriasis Patients with Biological Therapies in a Bucharest Hospital

**DOI:** 10.3390/jpm11080752

**Published:** 2021-07-30

**Authors:** Maria-Magdalena Constantin, Stefana Bucur, Costina-Cristiana Mutu, Elena Poenaru, Rodica Olteanu, Razvan Adrian Ionescu, Alin Codrut Nicolescu, Florentina Furtunescu, Traian Constantin

**Affiliations:** 1 Faculty of General Medicine, “Carol Davila” University of Medicine and Pharmacy, 050474 Bucharest, Romania; drmagdadinu@yahoo.com (M.-M.C.); elena.poenaru@gmail.com (E.P.); tane67@gmail.com (R.A.I.); florentina.furtunescu@umfcd.ro (F.F.); traianc29@yahoo.com (T.C.); 2 IInd Department of Dermatology, Colentina Clinical Hospital, 020125 Bucharest, Romania; cristiana.terziu@gmail.com (C.-C.M.); rodicaolteanu@hotmail.com (R.O.); 3 IIIrd Department of Internal Medicine, Rheumatology, Colentina Clinical Hospital, 020125 Bucharest, Romania; 4 Roma Medical Center for Diagnosis and Treatment, 011773 Bucharest, Romania; nicolescualin66@yahoo.com; 5 Department of Public Health and Management, 050463 Bucharest, Romania; 6 Department of Urology, “Prof. Dr. Th. Burghele” Hospital, 050659 Bucharest, Romania

**Keywords:** psoriasis, biological therapies, smoking status, clinical trial

## Abstract

Psoriasis is an immune-mediated chronic inflammatory skin disease with extracutaneous manifestations, that affects about 1–3% of the world’s population. The disease is not life-threatening, but the disability which comes with it is comparable to the disability caused by other serious chronic diseases, such as oncologic or cardiovascular disease. Several risk factors, such as infections, stress, smoking, excessive alcohol consumption and genetic predisposition have been involved in inducing psoriasis. Smoking status is a risk factor for many chronic diseases, including psoriasis. Moreover, recent studies have tried to answer the question of whether smoking also influences the response to biologic therapy in patients with psoriasis. Through the current study, our intention is to find out how smoking affects the response to biologic treatment. A hospital-based cross-sectional, observational, non-interventional, retrospective study of moderate and severe psoriasis patients receiving biologic treatment was developed. Two groups were defined based on smoking status: group 1 included smokers (more than 10 cigarettes/day) and former smokers, and group 2 included non-smokers. The data that resulted from the analysis of the cohort of patients demonstrate that smoking status does not affect the response of biologic therapy in patients with moderate and severe psoriasis.

## 1. Introduction

Psoriasis is an immune-mediated chronic inflammatory skin disease with extracutaneous manifestations that affects about 1–3% of the world’s population [1,2]. Smoking status is a risk factor for many chronic diseases, including psoriasis. Some studies have shown that cigarette smoking induces an overproduction of interleukin 1β (IL-1β) and increases the production of tumor necrosis factor α (TNF-α) and transforming growth factor β (TGF-β), which have been associated with the severity of the psoriasis [3] and also explains some of the associated comorbidities: cardiovascular disease, inflammatory bowel disease and several cancers [4]. Psoriasis is a papulosquamous disease with variable morphology, distribution, severity and course. There are different types and presentations of psoriasis and the most common form of the disease is plaque psoriasis [5].

It is well known that smoking influences the onset of the disease and also the evolution, but the effect of smoking on psoriatic patients being on biologic therapy is under debate. Some studies have suggested that smoking is associated with an increased severity of psoriasis and diminished treatment responses among patients with psoriasis [3,6].

Psoriasis is a chronic inflammatory disease that is mediated by Th1 and Th17 helper T cells. Smoking produces free radicals that may activate signaling pathways such as the mitogen-activated kinase, nuclear factor B and Janus kinase-STAT pathways. Byproducts from smoking, such as nicotine and dioxin, activate T cells that produce interleukin-12 (IL-12), interleukin-17 (IL-17) and interleukin-23 (IL-23), which are also involved in the pathogenesis of psoriasis [7,8]. Because IL-17 is one of the major cytokines involved in the pathogenesis of psoriasis, there are mechanistic reasons to believe that smoking could contribute to the development of psoriasis [9,10].

Plaque psoriasis can be classified as mild, moderate or severe on the basis of the degree of erythema, desquamation and infiltration, and the extent of body surface area involvement [11]. A wide variety of scoring systems have been proposed to evaluate severity in psoriasis [12]. In the study by Naldi et al. [13] who reviewed 171 randomized clinical trials published between 1997 and 2000, more than 40 scoring systems were identified. Evaluation of psoriasis severity is a multidimensional approach that takes into account several factors [14]. According to the literature, Psoriasis Area Severity Index (PASI) is a reliable, reproducible and responsive instrument and its internal consistency [15], intra-observer reliability [16,17,18] and inter-observer reliability [16,17,19] are significantly valid. PASI is one of the most used scores of disease severity in psoriasis and represents the first step in selection of the treatment.

The majority of patients with mild-to-moderate psoriasis is capable of adequately controlling the disease solely with topical medications or phototherapy. Biologic agents, administered as monotherapy or in combination with other topical or systemic drugs, have a high benefit-to-risk ratio and, due to this situation, are a welcome addition to the psoriasis management armament [20,21]. There are several types of biologic therapy, depending on their mechanism: TNF-α inhibitors, IL-12/23 inhibitor, IL-17 or IL-23 inhibitors.

## 2. Materials and Methods

A hospital-based cross-sectional, observational, non-interventional, retrospective study of moderate-to-severe psoriasis patients receiving biologic treatment was developed by the authors for which it received approval from the Ethics Committee. For requiring biologic therapy, patients must fulfill the inclusion criteria established in our country. A total of 109 patients from the Second Dermatology Department of the Colentina Clinical Hospital in Bucharest were enrolled in the study. These patients were included in the National Registry of Dermato-Venereological Diseases and signed the informed consent to participate in the study. These patients were treated with all types of biologic therapy available in our country: TNF-α inhibitors (etanercept (Pfizer, Brooklyn, NY, USA), adalimumab (Abbvie, Lake Bluff, IL, USA), infliximab (MSD, Wellington, New Zealand) and their biosimilars); IL-12/23 inhibitor (ustekinumab (Janssen, Beerse, Belgium)) and IL-17 inhibitors (secukinumab (Novartis, Basel, Switzerland), ixekizumab (Eli Lilly, Indianapolis, IN， USA)).

Detailed information about smoking history as well as the clinical severity of psoriasis were noted at baseline and one year after treatment initiation. The clinical severity of psoriasis was assessed using PASI score and the smoking history was assessed using a questionnaire of smoking habits. The Dermatology Life Quality Index score (DLQI) was used to quantify the impact of the disease on life quality. Two groups were defined based on the smoking status: group 1 included smokers (more than 10 cigarettes/day) and former smokers and group 2 included non-smokers. Smoking status included three categories: have never smoked, current smoker and former smoker. The latter includes those who had stopped smoking at least one year before being interviewed for this study. We used the Statistical Package for the Social Sciences (SPSS) software version 23 for Windows program. used for statistical data analysis. In order to determine the statistical significance we used Paired Samples T Tests and Chi-Square Tests.

Most patients in the study group have at least one comorbidity. These range from psoriatic arthritis, which is the most common, to metabolic syndrome, cardiovascular disease, obesity and diabetes mellitus. However, these variables were not followed in the current study.

Inclusion criteria were: evidence of a personally signed and dated informed consent form indicating that the subject has been informed of all pertinent aspects of the study; adult patients aged ≥18 years at the time of screening; clinical diagnosis of psoriasis and anatomopathological diagnosis confirmation; diagnosis of severe psoriasis defined as PASI > 10; eligible for biologic treatment according to the National Protocol for Biological Therapy in psoriasis and to Summary of Product Characteristics; biologic-naive patients; smokers who smoke >10 cigarettes daily for smokers group (group 1) or non-smokers for non-smoking group (group 2); capable of understanding and completing questionnaires.

Exclusion criteria were: failure to adequately cooperate in the study; hypersensitivity to the active substances or to any of the excipients; active infections, sepsis or risk of sepsis; positive pregnancy test, breast feeding or considering becoming pregnant during the study; significant drug or alcohol abuse.

The objectives of the study were to determine the percentage of patients achieving a PASI 100 response (complete remission) in group 1 versus group 2 after one year of biologic therapy, to determine the quality of life among enrolled patients after one year of biologic therapy and to evaluate the impact of smoking over the treatment outcome.

## 3. Results

The study reported data obtained from 46 females and 63 males, with the mean age of 52.59 for females and 50.06 for males. From the total of 109 patients, 68 patients (62.38%) of the group live in the urban areas and 41 patients (37.61%) come from rural areas. It was observed that the percentage of smoking patients of the total number of patients in the study was 46.8%. Former smokers accounted for 7.3% and non-smokers accounted for 45.9%, which means that group 1 included 54.1% of patients and group 2 included 45.9% of patients.

The percentages of patients who obtained complete remission one year after the initiation of biologic therapy were 50.2% in group 1, respectively 49.2% in group 2.

**Figure 1:**
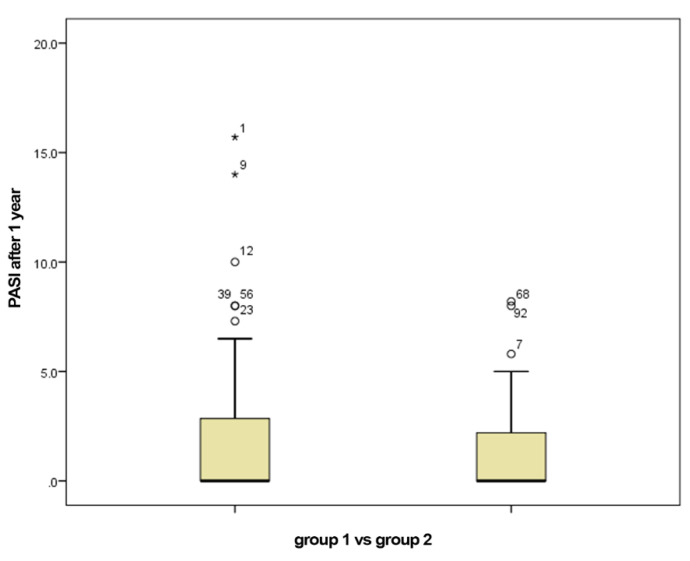
PASI after 1 year of biological therapy for group 1 (smokers) vs. group 2 (non-smokers).

The mean PASI at the initiation with biological therapy in group 1 was 19.87, with a median of 20.15, compared to the mean PASI of 20.66 in group 2, with a median of 21. The mean PASI after one year of biologic therapy in group 1 was 2.20, with a median of 0, compared to 1.29, the mean PASI after one year of biologic therapy in group 2, with a median of 0 (Figure 1).

To evaluate the clinical response to biologic therapy, a variable called DIFFPASI was calculated. DIFFPASI represents the difference between the initial PASI and PASI after one year of biologic therapy. The value of this variable is 18.44 in group 1, which means that the PASI score decreased by an average of 18.44 in group 1. The value of DIFFPASI is 18.57 in group 2, which means that PASI score decreased by an average of 18.57 in the non-smokers group (Table 1). *p* value is 0.921 (>0.05), which means that there is no statistically significant difference between the two groups.

**Figure 2:**
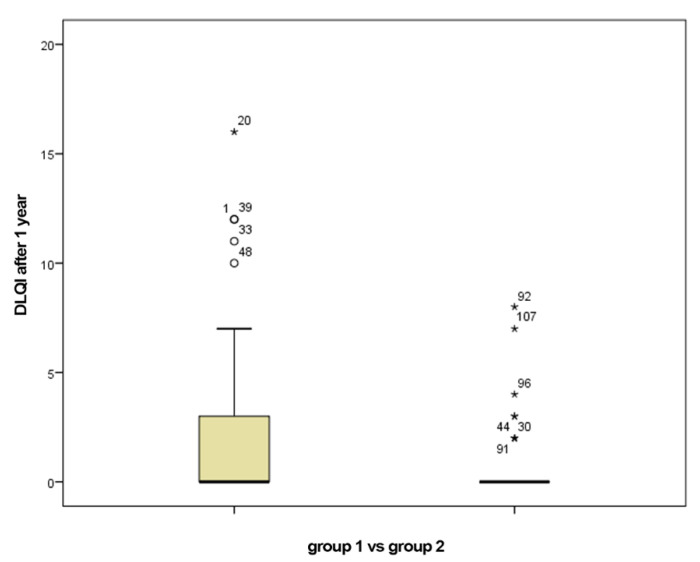
DLQI after 1 year of biological therapy for group 1 (smokers) vs. group 2 (non-smokers).

The percentages of patients who obtained a DLQI score of 0 after one year from the initiation of biologic therapy are 44.4 for group 1 and 55.6 for group 2, respectively. Regarding the DLQI score, it had a value of 22.3 at baseline with a median at 22.5 in group 1 versus the value of 22.3 with a median of 22 in group 2. The mean value of DLQI score one year after initiation of biologic therapy in group 1 was 2.22 with a median of 0 compared with the mean value of DLQI score of 0.7 with a median of 0 in group 2 (Figure 2). To compare the DLQI scores in the two groups, we defined the DIFFDLQI variable, which represents the difference between the baseline DLQI and the DLQI score after one year of biologic therapy. For group 1 the average value of this variable was 20.15 and for group 2 the average value was 21.60 (Table 2). *p* value was 0.09 (>0.05), which means that there was no statistically significant difference between the two groups.

**Figure 3:**
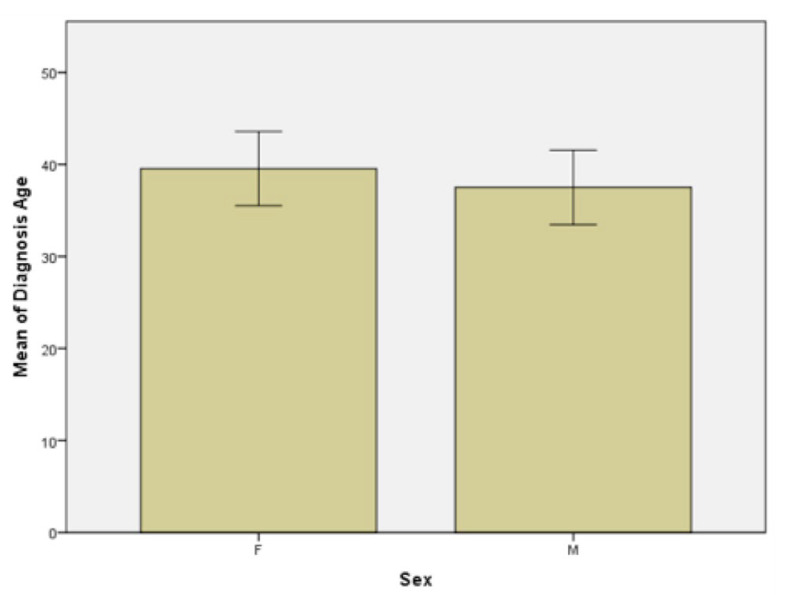
Age at psoriasis diagnosis in females (F) and males (M) subgroups.

Group 1 and group 2 were each divided into two subgroups by gender: females and males. Other data that emerged from the statistical analysis of the two subgroups are the following: firstly, the age at which the disease was diagnosed in the women subgroup ranged from 3 years to 64 years, with a median of 38.5 years and a mean age of 39.5 and the age at which the disease was diagnosed in the men subgroup ranged from 7 years to 75 years, with a median of 35 years and the mean age of 37.5 (Figure 3). In the women subgroup, the histogram is asymmetric on the left side, compared to the asymmetric histogram on the right side in the men subgroup, which suggests the later onset of the disease in the female population. In our study a gender difference was found in the age of onset of psoriasis, but regardless of gender, most patients developed psoriasis before the age of 40, the data being in accordance with those reported by Queiro et al. [22].

**Figure 4:**
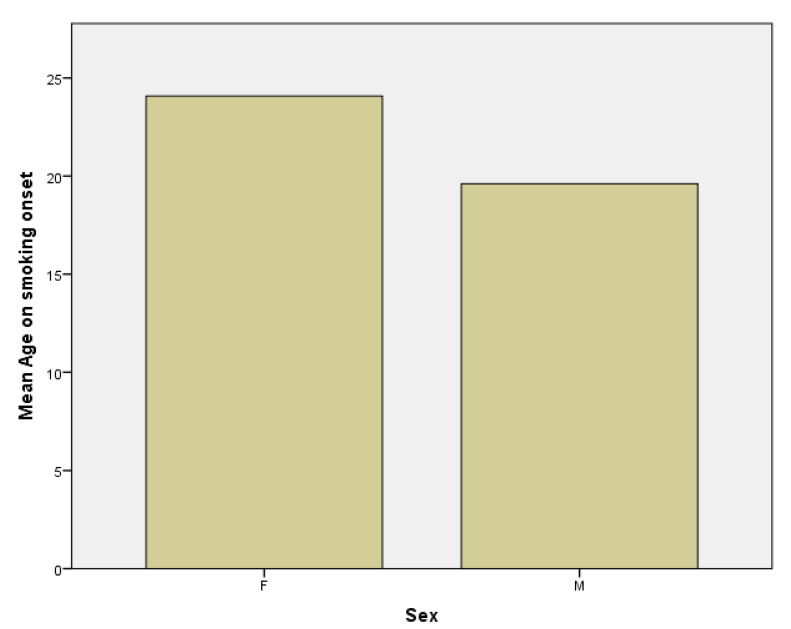
Age at smoking onset in females (F) and males (M) subgroups.

Secondly, the mean age in women subgroup at which patients started smoking is 24.08 years, with a median of 21.5 years compared to the mean age in the men subgroup, which is 19.61 with a median of 18 years (Figure 4). This means that the onset of smoking is earlier in male population and the difference is statistically significant with a *p* value of 0.02 (*p* < 0.05).

**Figure 5:**
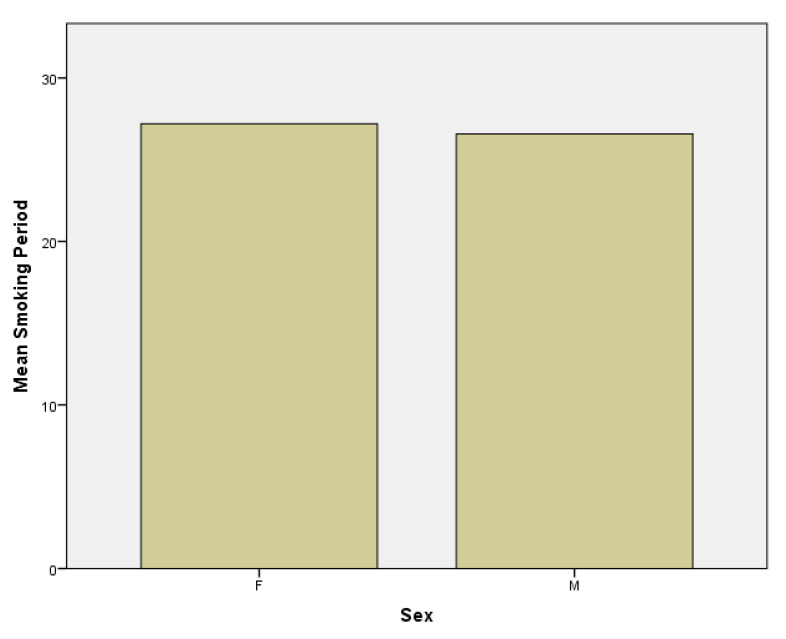
Smoking period in females (F) and males (M) subgroups.

Thirdly, regarding the smoking period, in the female subgroup the mean smoking period is 27.19 years versus 26.58 years in the male subgroup (Figure 5).

**Figure 6:**
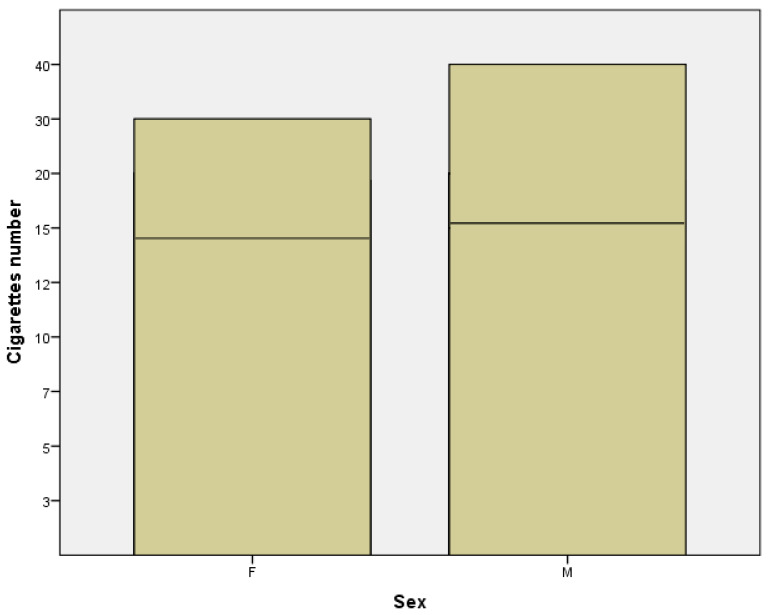
Number of cigarettes in females (F) and males (M) subgroups.

Another result that emerged was that the average number of cigarettes smoked per day was 14.42 in women subgroup compared to 16.52 in men subgroup (Figure 6). From the last three obtained data, we can affirm that men smoke a shorter period of time than women, but they counterbalance regarding the higher number of cigarettes smoked per day. This conclusion is supported by several data from literature which report that the average number of cigarettes smoked daily is higher among males than females [23].

## 4. Discussions

Psoriasis is one of the many diseases associated with smoking and patients may be more motivated to consider quitting because of it than in other invisible skin health problems. The harmful influence of smoking is related to the induction of inflammatory mediators involved in the pathogenic phenomena of the skin of patients with psoriasis. Questioning smoking in psoriasis patients and supporting smoking cessation can help reduce the impact of smoking on psoriasis.

Our cohort was balanced, with a comparable number of patients in the two groups. Regardless of the smoking status, all the patients suffered from moderate or severe psoriasis.

In this study, the gender difference in the age of onset of psoriasis revealed an earlier onset of the disease in the male population. However, most patients developed psoriasis before the age of 40. In addition, in the male population, the onset of smoking was statistically significant earlier, compared to the female population, but men smoke for a shorter period of time than women; this was a counterbalance in terms of the higher number of cigarettes smoked per day by the female population.

The data resulted from the analysis of this cohort demonstrate that smoking status does not affect the response of the biologic therapy in patients with moderate and severe psoriasis and similar results were obtained in many studies [24,25,26,27,28,29]. Although evidence is not strong, some reports indicate no association between smoking status and the PASI 75 response rate to adalimumab or other biologic therapies [29]. Another study shows that no difference was found between smoking status and the effectiveness of TNF-α blockers and ustekinumab in a retrospective Italian study of 350 patients [30].

Currently, there is no consensus on the impact of smoking on the therapeutic response in patients with psoriasis, which means firstly that medical data are insufficient and secondly reveals the difficulty of this topic. Numerous meta-analyzes have tried to elucidate this unknown fact, but the final results have not been consistent. On the one hand, there are authors who reported that smoking influences the therapeutic response in patients and postulated that smokers were less likely to show disease improvement in the disease at 6 months after treatment with biologic agents than non-smokers [1], and, on the other hand, there have been studies suggesting that active smoking does not significantly affect the response to treatment [28].

A recent meta-analysis published in 2020 suggests that the number of reports is limited and more studies are needed to confirm the effects of smoking and smoking cessation on the therapeutic response in patients with psoriasis [1].

Medical data is limited on this subject and more studies are needed to establish the effects of smoking regarding the therapeutic response in patients with moderate and severe psoriasis in treatment with biologic therapy.

However, the harmful effects of smoking are undeniable. These are found and can influence both the etiopathogenesis of psoriatic disease and the evolution, severity and treatment of this condition. In this study we tried to evaluate the effects of smoking on the group of patients in our hospital.

## 5. Conclusions

The data that resulted from the analysis of the cohort of patients demonstrate that smoking status does not affect the response of biologic therapy in patients with moderate and severe psoriasis.

Regardless of how smoking influences the therapeutic response of patients with biological therapy, it is clearly proven that smoking is involved in the pathogenesis of psoriasis, both as a trigger for the disease and as an aggravating factor. Thus, doctors have the duty to advise and encourage the patient to quit or reduce smoking, this being an essential step in the management of the disease.

This result obtained in our study is sustained by many studies in the specialized literature. However, more studies with as many patients as possible are needed to reach a unanimous final conclusion.

## Figures and Tables

**Table 1:** DIFFPASI in group statistics.

	Smokers vs. Non-Smokers	N	Mean	Std. Deviation	Std. Error Mean
DIFFPASI	Group 1	59	−18.4458	8.20268	1.06790
	Group 2	50	−18.5780	5.55534	0.78564

**Table 2:** DIFFDLQI in group statistics.

	Smokers vs. Non-Smokers	N	Mean	Std. Deviation	Std. Error Mean
DIFFDLQI	0	59	−20.1525	6.37564	0.83004
	1	50	−21.6000	4.30946	0.60945

## Data Availability

Data from this study are available at the Second Dermatology Department of the Colentina Clinical Hospital in Bucharest and will be made available upon request.
